# Mid-infrared sensing of CO at saturated absorption conditions using intracavity quartz-enhanced photoacoustic spectroscopy

**DOI:** 10.1007/s00340-019-7260-6

**Published:** 2019-08-05

**Authors:** Jakob Hayden, Bettina Baumgartner, Johannes P. Waclawek, Bernhard Lendl

**Affiliations:** Institute of Chemical Technologies and Analytics, Technische Universität Wien, Getreidemarkt 9/164, 1060 Vienna, Austria

## Abstract

The sensitivity of quartz-enhanced photoacoustic spectroscopy (QEPAS) can be drastically increased using the power enhancement in high-finesse cavities. Here, low noise resonant power enhancement to 6.3 W was achieved in a linear Brewster window cavity by exploiting optical feedback locking of a quantum cascade laser. The high intracavity intensity of up to 73 W mm^−2^ in between the prongs of a custom tuning fork resulted in strong optical saturation of CO at 4.59 µm. Saturated absorption is discussed theoretically and experimentally for photoacoustic measurements in general and intracavity QEPAS (I-QEPAS) in particular. The saturation intensity of CO’s R9 transition was retrieved from power-dependent I-QEPAS signals. This allowed for sensing CO independently from varying degrees of saturation caused by absorption induced changes of intracavity power. Figures of merit of the I-QEPAS setup for sensing of CO and H_2_O are compared to standard wavelength modulation QEPAS without cavity enhancement. For H_2_O, the sensitivity was increased by a factor of 230, practically identical to the power enhancement, while the sensitivity gain for CO detection was limited to 57 by optical saturation.

## Introduction

Besides tunable laser absorption spectroscopy (TLAS), photoacoustic spectroscopy (PAS) is the most widely employed laser spectroscopic technique for trace gas sensing [1]. While direct absorption spectroscopy, such as TLAS, measures the attenuation of light, PAS measures sound waves generated locally in the sample by the absorbed power (indirect detection). From an application perspective, the biggest advantage of PAS as compared to TLAS is that it allows measuring in very small gas volumes of typically a few cm^3^ or even well below without losing sensitivity with decreasing size. Although ppm and ppb level sensitivities are routinely achieved, the detection limits of PAS are still higher than those of direct absorption spectroscopy, such as TLAS. Reported sensitivities of PAS, typically given as noise equivalent absorption coefficient (NNEA), normalized by the bandwidth of the measurement and inverse laser power, vary between different molecules and typically range from 10^−8^ cm^−1^ W Hz^−1/2^ (CO, [2, 3]) to 10^−9^ cm^−1^ W Hz^−1/2^ (H_2_O, C_2_H_2_, [4]). For laser powers of 20 mW to 100 mW, representing typical values for quantum cascade lasers (QCL) commonly employed in the mid-infrared (IR) range, this corresponds to noise equivalent absorption coefficients (NEA) of 10^−8^ cm^−1^ Hz^−1/2^ and above. On the contrary, direct absorption spectroscopic sensing is now commonly performed in the mid-IR range well below 10^−9^ cm^−1^Hz^−1/2^ using multipass absorption cells [5, 6] or even below 10^−10^ cm^−1^ Hz^−1/2^ using different techniques of cavity-enhanced spectroscopy [7–9]. Continued efforts are taken to exploit the advantages of PAS while, at the same time, achieving or exceeding the sensitivity of TLAS, e.g. by improving acoustic transducers [10] or by investigating novel approaches to PAS and other techniques of indirect spectroscopy [11, 12].

To enhance the sensitivity of PAS, a straight forward way is to increase the optical power *P*, since the photoacoustic signal *S* is directly proportional to *P*,

1 $$\begin{array}{*{20}c} {S = kP\alpha .} \\ \end{array}$$

Herein, *α* is the absorption coefficient (to base e) and *k* is a constant representing the photoacoustic response to absorbed optical power. High levels of optical power can be achieved using high power lasers [3], by performing PAS inside a laser resonator [13–15] and by employing the power enhancement of light coupled into an external high finesse cavity [16–18]. The buildup of optical power inside an optical cavity was demonstrated to increase sensitivities by factors up to 240 [19] in the mid-IR and 630 in the near-IR [20], yielding a NEA < 10^−9^ cm^−1^ Hz^−1/2^ [17].

While the large majority of previous studies confirmed the direct proportionality between *S* and *P*, very high *P* eventually leads to saturated absorption and a non-linear scaling [13, 21, 22]. Saturated absorption, i.e. a decrease of *α* with increasing optical intensity, is observed when the rate of excitation approaches or is bigger than the rate of relaxation, hence less molecules are available for excitation. For PAS in the mid-IR, excitation rates are often high since (a) absorption cross-sections of fundamental ro-vibrational transitions are large (as compared to, e.g. those in the near-IR), and (b) the laser beam is typically tightly focused in the sample, yielding high intensities (as opposed to TLAS). At pressures relevant to PAS, relaxation from vibrationally excited states is predominantly facilitated by molecular collisions. Relaxation rates vary by orders of magnitude between different molecules and collision partners [23]. For example, one out of ~ 20,000, 1300 and 40 collisions of CO with N_2_, H_2_O and N_2_O, respectively, leads to vibrational relaxation of CO [23, 24].

Saturated absorption was discussed in the context of cavity ring-down spectroscopy (CRDS) [25] and was even advantageously utilized for ultrasensitive CRDS sensing [26]. For PAS, saturated absorption was reported in a number of publications and sensitive measurements were demonstrated in this regime [21, 22, 27]. Although operating in a saturated regime, linearity with the concentration of the target gas is still given as long as the optical power is not significantly reduced by absorption of the gas, which usually is the case in PAS [21]. On the contrary, the power inside high finesse cavities decreases with increasing absorption [28, 29]. Hence, a quantitative treatment of saturated absorption PAS is necessary to fully exploit the sensitivity enhancement provided by high finesse cavities.

In this contribution, the influence of saturated absorption on intracavity PAS is quantitatively discussed both theoretically and experimentally. We present intracavity quartz enhanced photoacoustic spectroscopy (I-QEPAS) measurements of CO under highly saturated conditions targeting the fundamental R9 ro-vibrational transition of CO at 2179.77 cm^−1^. The power of 25 mW from a QCL was enhanced to 6.3 W inside a linear high finesse cavity. Efficient and low noise intracavity power buildup was achieved by exploiting optical feedback from a Brewster window placed inside the cavity [30]. The optical setup and its operation are described in detail. Saturation broadening of the targeted absorption profile was observed in a spectrum of ambient air at 250 mbar, clearly indicating strong saturation. The degree of saturation was quantified by retrieving the saturation intensity from power-dependent measurements at 200 mbar and 500 mbar. A calibration of CO is presented that shows clear deviations from linearity at high concentrations associated with saturated absorption. Linearization is performed using the measured saturation intensity and theory of saturated absorption. This allows taking full advantage of the power enhancement in I-QEPAS beyond the linear absorption regime. This is demonstrated by sensing single-digit ppb concentrations of CO in a saturated regime. The obtained figures of merit are compared with standard 2*f*-wavelength modulation QEPAS (2*f*-WM QEPAS) measurements without cavity enhancement.

## Experimental

### Setup [31]

**Fig. 1:**
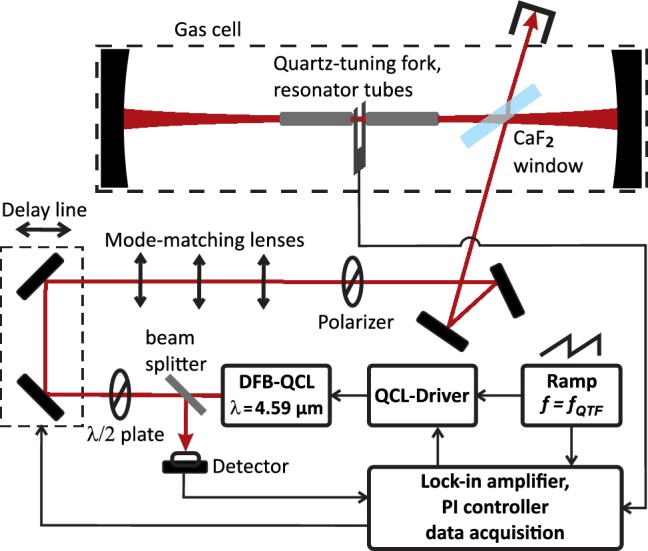
Experimental setup, detailed description see text

The setup is depicted schematically in Fig. 1. A thermo-electrically cooled DFB-QCL (AdTech Optics) emitted 45 mW of optical power in continuous-wave operation at a wavelength of 4.59 µm. A half-wave plate and polarizer set the polarization to parallel with respect to the CaF_2_ window in the cavity. Three plano-spherical lenses of focal lengths 200 mm, − 200 mm and 150 mm were used for mode matching to the cavity’s fundamental Gaussian mode. The cavity mirrors (Layertec) had a radius of curvature (ROC) of 150 mm and a measured reflectivity of 0.9992 (compare Sect. 2.3). The cavity length was 0.28 m and could be fine-tuned using a piezo-electrically actuated cavity mirror mount (Thorlabs, KC1-PZ/M) to vary the cavity’s resonance frequency over a full free spectral range (FSR). The small ROC and close to concentric cavity design were chosen to achieve a waist of the fundamental cavity mode much smaller than the prong spacing of the quartz tuning fork (QTF). A CaF_2_ window of 2 mm thickness was positioned in the cavity close to Brewster’s angle (*θ*_B_ = 54.5° at 4.59 µm, compare Sect. 2.3) using a compact rotation mount (Thorlabs, FBTP). Light was coupled into the cavity via reflection at the Brewster window’s front surface [30]. A thermo-electrically cooled mercury-cadmium-telluride photodetector (PCI-2TE-12, Vigo Systems S.A.) detected a small fraction (~ 1%) of the light reflected back from the cavity to the laser picked up via a beam splitter. Two mirrors on a piezo actuated stage were used as a delay line to fine-tune the phase of light back-reflected into the laser (compare Sect. 2.2). A QTF and resonator tubes (length = 12.45 mm, inner diameter = 1.5 mm) were positioned in the center of the cavity using a compact five-axis stage (Thorlabs, PY005/M). The QTF was a custom design for QEPAS with a resonance frequency of 15.82 kHz and a prong spacing of 1.5 mm (see [32], design S15). It was provided in a ready to use module (Thorlabs, prototype of ADM01) including electronic amplification and excitation circuitry by Thorlabs GmbH. The use of the resonator tubes yielded a QEPAS signal-to-noise enhancement by a factor of six as compared to the bare tuning fork. The amplified signal from the tuning fork was demodulated using a lock-in amplifier (Zurich Instruments, MFLI) and normalized by the photo-detector signal, which was demodulated at the same frequency using the same lock-in amplifier. The detector signal recording the back reflection from the cavity is proportional to intracavity power and was used to normalize the recorded QEPAS signal to optical power. The optical cavity as well as QEPAS module and Brewster window were enclosed in an aluminum gas cell (volume = 1.7 L) with CaF_2_ windows for in- and out coupling of the laser beam. The full optical setup was enclosed in a simple housing to reduce the impact of turbulent airflow on the optical feedback phase.

### Principles of operation

**Fig. 2:**
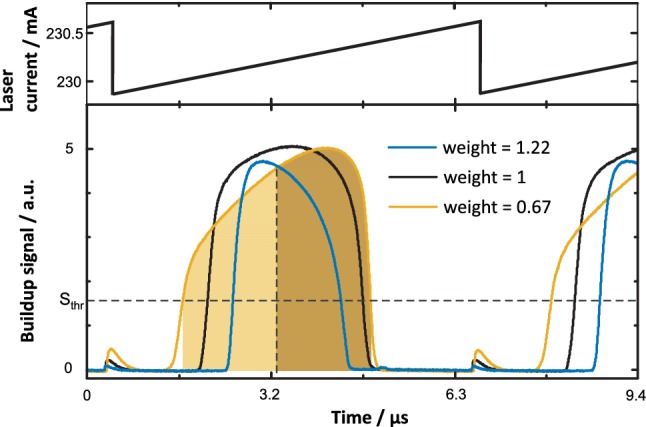
Cavity buildups recorded during ramped current modulation of the QCL. The three lines represent different optical feedback phases, corresponding to different “weights” of the buildups. The feedback phase is detected and corrected based on weighting of the first and second half of the buildups (shaded areas, see text). Incomplete buildups observed during the falling edge of current modulation do not interfere with I-QEPAS measurements

To generate photoacoustic signals inside the cavity, the optical power inside the cavity was modulated at the tuning fork’s resonance frequency *f*_QTF_. Efficient and low-noise modulation of intracavity power was achieved by exploiting optical feedback locking [16, 17]. The detector signal, which is proportional to the intracavity power, during a period of laser modulation is shown in Fig. 2. The QCL’s wavelength was modulated via the driving current using a small amplitude ramping signal applied to the analog control input of the QCL-driver (Wavelength electronics, QCL-OEM 300). Starting at *t* = 0 in Fig. 2, the free running laser wavelength $\lambda_{\text{free}}$ is initially detuned from the cavity resonance wavelength $\lambda_{\text{res}}$. As $\lambda_{\text{free}}$ approaches $\lambda_{\text{res}}$, some fraction of the incident light resonates in the cavity and is reflected back into the laser, i.e. optical feedback occurs [28]. The response of the QCL to optical feedback depends, amongst others, on the phase of the back-reflected light [33], which was adjusted via the physical length between the laser and the cavity using a delay line (compare Fig. 1). If the back-reflection is in phase with the emitted light, optical feedback causes a dramatic stabilization of the emitted wavelength to a wavelength closely matching $\lambda_{\text{res}}$. This process leads to highly efficient coupling of light into the cavity and accordingly large buildup of optical power. As $\lambda_{\text{free}}$ is detuned from $\lambda_{\text{res}}$ far enough during the modulation, the QCL’s wavelength returns to $\lambda_{\text{free}}$ and no buildup occurs. A cycle of modulation is completed by swiftly shifting $\lambda_{\text{free}}$ to the opposite side of $\lambda_{\text{res}}$. Experimentally, ramped modulation was preferred over sinusoidal or triangular modulation at *f*_QTF_/2 [17] since, generally, the observed buildup was not symmetric with respect to scan direction. The stability of buildups was best for the chosen scan direction and when the feedback phase was adjusted for slightly asymmetric buildups (weight < 1, compare Fig. 2).

To record QEPAS spectra at increments of the cavity’s FSR, the same feedback phase must be achieved for all consecutive resonances of the cavity. To this end, the distance between laser and cavity was adjusted to a multiple of the cavity length [28]. To stabilize the feedback phase in wavelength locked operation, a number of methods based on the shape of the buildup signal were demonstrated [34–36] (compare Fig. 2). Here, the detector signal was digitized and analyzed in a real-time application programmed in Matlab on a personal computer. Each buildup was isolated based on a signal threshold level (*S*_thr_ in Fig. 2) and the first and second half (by number of samples) of the buildup were integrated (shaded areas in Fig. 2). The error signal for the proportional-integral (PI) algorithm was obtained by division of the areas under the first half and the second half of the buildups. The set point of the so obtained “weight” of the buildups was chosen as 0.82. The feedback phase was adjusted by excursion of the delay line using the analog control input of the piezo controller (Physik Instrumente, E-536.30).

The detector signal shown in Fig. 2 also served for locking the laser center wavelength to a cavity resonance based on a standard wavelength modulation approach [37]. To this end, the signal was demodulated at the first harmonic of the modulation frequency using the lock-in amplifier. A digital laser locking module (Toptica Photonics, Digilock 110) hosted a PI controller that regulated the in-phase component of the demodulated signal to zero by acting on the laser current via the analog control input of the QCL driver.

For measurements of cavity losses discussed in Sect. 2.3, the setup also allowed performing cavity ring-down spectroscopy (CRDS). To record ring-downs, the laser current was swiftly reduced below the threshold current using the analog control input of the QCL driver. The detector signal was digitized with a vertical resolution of 8 bit using an oscilloscope (LeCroy, Waverunner 64 Xi). The initiation of ring-downs was synchronized to a ramped current modulation (compare Fig. 2) at 500 Hz independent from a threshold detector signal commonly used in CRDS [30]. The repetition rate of ring-downs (500 Hz) was kept low compared to the inverse down time of ring-down events (0.5% duty cycle) to limit the impact of the reduced heat load on the laser wavelength. Each ring-down was fitted in post processing using a three parameter single exponential model and the fitted ring-down times were averaged.

To evaluate the gain in sensitivity achieved through the optical cavity, measurements of CO and H_2_O were referenced against (2*f*-WM QEPAS). To this end, the QEPAS module was removed from the optical cavity and the beam was guided through the resonator tubes and prongs of the tuning fork. For measurements of CO, a reference gas cell containing CO was used to lock the laser wavelength to the CO resonance. The third harmonic of the signal from the photo-detector recording the transmission through the reference cell was used for this purpose [37].

### Optical impedance matching

To maximize the gain in sensitivity, the intracavity power buildup was maximized. The optical power that builds up inside a cavity on resonance depends not only on the cavity losses but also on the transmission of the mirror through which light is coupled into the cavity. For the Brewster window cavity, rather than the transmission of the in-coupling mirror, the reflectivity *R*_B_ of the Brewster window determines how much light is coupled into the cavity. *R*_B_ and hence the power buildup can be adjusted conveniently by changing the angle at which the window is placed in the cavity, which is a unique feature of the Brewster cavity design [31, 38]. The bidirectional power *P*_intra_ circulating inside the cavity on resonance assuming perfect mode matching as a function of *R*_B_ is given by (see Appendix A for derivation)

2 $$\begin{array}{*{20}c} {P_{\text{intra}} = P_{0} \frac{{2R_{\text{B}} R_{\text{M}} }}{{\left( {1 - R_{\text{M}} + 2R_{\text{B}} + \alpha L} \right)^{2} }}.} \\ \end{array}$$

Herein, *P*_0_ is the incident laser power, *R*_M_ is the mirror reflectivity (assumed equal for both mirrors) and *αL* are the combined half round-trip losses excluding reflective losses. The largest buildup $\frac{{P_{\text{intra}} }}{{P_{0} }} = \frac{1}{4}\frac{{R_{\text{M}} }}{{\left( {1 - R_{\text{M}} + \alpha L} \right)}}$ is found for $R_{\text{B}} = \frac{1}{2}\left( {1 - R_{\text{M}} + \alpha L} \right)$, i.e. for *R*_B_ matching half the combined half round-trip losses of all other elements in the cavity. Note that the buildup can be increased by a factor of two by eliminating the reflective losses at the rear surface of the Brewster window. This can be achieved using an appropriately wedged window that puts the rear surface exactly to Brewster’s angle. A discussion on the properties of the Brewster window cavity relevant to PAS is given in Appendix B.

The mirror and cavity losses ($1 - R_{\text{M}} + \alpha L$) were unknown when the mirrors were received and could not be measured using CRDS without the Brewster window since the mirrors were deposited on non-transparent substrates, preventing coupling into the cavity through the mirrors. Therefore, the losses $1 - R_{\text{M}} + \alpha L$ were retrieved from measurements of *P*_intra_ and ring-down time *τ* at different angles of the Brewster window. *P*_intra_ was monitored by recording the I-QEPAS signal from a water line at 2178.9 cm^−1^ from ambient air at atmospheric pressure and normalization of signals to humidity recorded separately. *τ* is related to the cavity losses as:

3 $$\begin{array}{*{20}c} {\frac{1}{\tau } = \frac{c}{2L}\left( {1 - R_{\text{M}} + \alpha L + 2R_{\text{B}} } \right)} \\ \end{array} .$$

**Fig. 3:**
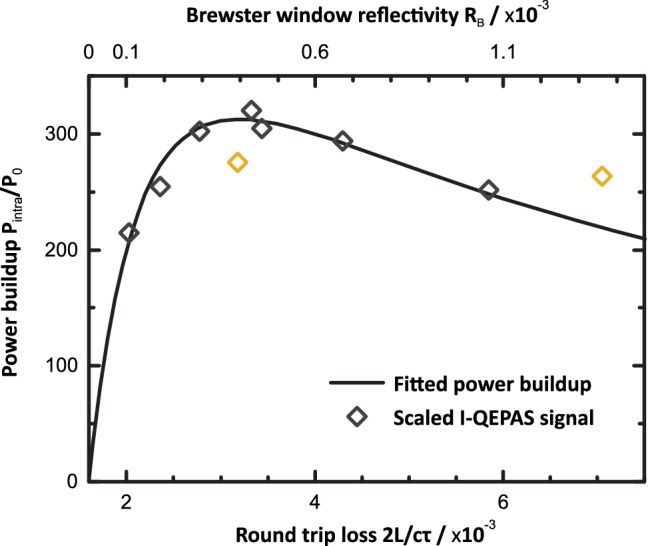
Intracavity power buildup [calculated assuming perfect mode matching, see (2)] and scaled I-QEPAS signal from water (measured, outliers marked yellow) versus Brewster window reflectivity. Adjusting the reflectivity of the Brewster window via its angle allows maximizing the intracavity power buildup

Solving (3) for *R*_*B*_ and inserting in (2) allows fitting of the I-QEPAS signal recorded for different *τ* using the combined losses $\ell = 1 - R_{M} + \alpha L$ and *P*_*0*_ as fitting parameters. The recorded I-QEPAS signal as well as the fit of (2) and corresponding power buildup *P*_intra_/*P*_0_ are shown in Fig. 3. Two outliers indicated in Fig. 3 were excluded for the fitting. The retrieved combined losses (half round-trip) are $\ell = 8 \times 10^{ - 4}$. At the optimized angle of the Brewster window of 52.2° (*Θ*_B_ = 54.54°), *P*_intra_/*P*_0_ = 312. For this angle, the cavity finesse is 2000.

**Fig. 4:**
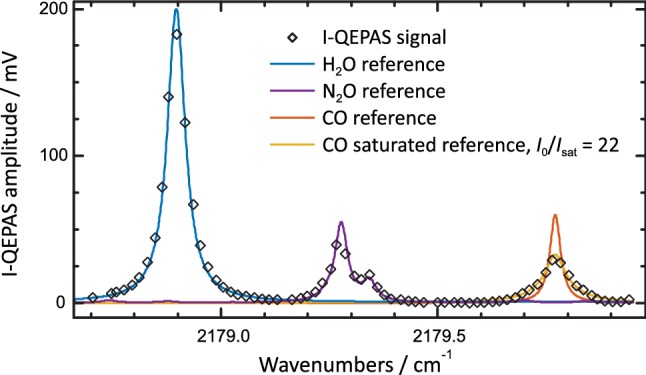
I-QEPAS spectrum of ambient air at *p* = 250 mbar. Scaled simulated absorption spectra of H_2_O and N_2_O nicely resemble the measured I-QEPAS spectrum. The CO absorption line at 2179.77 cm^−1^ is significantly broadened as compared to the reference absorption spectrum due to saturation broadening. Observed peak absorption coefficients of H_2_O and CO estimated from ambient concentration levels are ~ 3 × 10^−6^ cm^−1^ and 7 × 10^−6^ cm^−1^

Since perfect mode-matching as assumed in (2) was not achieved, the actual power buildup is lower than given in Fig. 3 [39]. To measure the mode-matching efficiency and effective intracavity power at maximum buildup, the cavity transmission *T*_Brewster_ on resonance was measured using a power meter placed behind the Brewster window (position of beam dump in Fig. 1). The measurement was compared to the theoretically expected transmission (compare Appendix A),

4 $$\begin{array}{*{20}c} {T_{\text{Brewster}} = \frac{{\left( {1 - R + \alpha L + R_{\text{B}} } \right)^{2} }}{{\left( {1 - R + \alpha L + 2R_{\text{B}} } \right)^{2} }}} \\ \end{array}$$

which equals *T*_Brewster_ = 9/16 for the impedance matched cavity. The ratio $\frac{{1 - T_{\text{Brewster,measured}} }}{{1 - T_{\text{Brewster}} }}$ gives a coupling efficiency of 81% and, with an incident laser power of 25 mW on the Brewster window, an intracavity power of $P_{\text{intra}} = 0.025\; {\text{W}} \times 312 \times 0.81 = 6.3\; {\text{W}}$. All measurements presented below, except for the I-QEPAS spectrum shown in Fig. 4, were recorded with optimized angle and *P*_intra_ = 6.3 W, corresponding to a peak intensity *I*_0_ = 73 W mm^−2^ at the center of the cavity [compare (6)]. For the data in Fig. 4, *P*_intra_ = 2.2 W, corresponding to *I*_0_ = 26 W mm^−2^.

### Measurements and sample preparation

I-QEPAS spectra were recorded by stepping the laser wavelength via temperature in increments of the cavity’s FSR. The laser wavelength and optical feedback phase were locked for each cavity resonance and signals were recorded for a given time (see below). For all other measurements, the laser wavelength was locked to a cavity resonance and the resonance wavelength was tuned to the absorption peak via the piezo-driven cavity mirror mount.

All measurements presented herein were performed in a steady flow of sample gas at flow rates below 500 sccm at ambient temperature. Samples of known concentration of CO were prepared from test gas (Air Liquide) of 952 ppb and 100 ppm, respectively, of CO in N_2_ using a custom-built gas mixing unit based on mass flow controllers. With the exception of ambient air, all samples discussed below were humidified to a water concentration of 1.7%_V_ by mixing the dry test gas with humidified nitrogen (grade 5.0) to enhance photoacoustic signals. The latter was prepared by bubbling nitrogen through the water at a stabilized temperature of 22 °C. The humidity of the sample gas was measured using a thermo-hygrometer (Testo, 635). A discussion on the influence of water on photoacoustic signals, in particular those from CO, can be found in [3, 40, 41].

## Results and discussion

### Spectrum of ambient air

An I-QEPAS spectrum of ambient air at a pressure of 250 mbar is shown in Fig. 4. The wavelength was stepped in increments of the cavity’s FSR (0.018 cm^−1^) and the I-QEPAS signal was recorded for 30 s per data point. Absorption lines of CO, H_2_O and N_2_O are observed.

The I-QEPAS spectrum matches scaled simulated reference spectra [42] of H_2_O and N_2_O. On the contrary, the CO band at 2179.77 cm^−1^ is much broader than the simulated absorption profile. As shown in Sect. 3.2, the line broadening can be attributed to optical saturation (saturation broadening). The degree of saturation can be estimated from the recorded line profile using (5). The saturated absorption profile $\alpha \left( {\nu - \nu_{0} } \right)$ of a homogeneously broadened transition in a collimated Gaussian beam is described by [43]:

5 $$\begin{array}{*{20}c} {\alpha = \alpha_{0} \frac{{I_{\text{sat}} }}{{I_{0} }}\ln \left( {1 + \frac{{I_{0} }}{{I_{\text{sat}} }}V\left( {\nu - \nu_{0} } \right)} \right).} \\ \end{array}$$

Herein, *α*_0_ is the linear absorption coefficient at resonance, *I*_0_ is the peak intensity of the Gaussian intensity profile, *I*_*sat*_ is the saturation intensity and *V*(*ν *− *ν*_0_) is the peak normalized line-shape function. Note that (5) is valid only if the Gaussian beam does not diverge along the probed distance, which is the case at the position of the QTF in the center of the cavity. Equation (5) can be generalized by inserting *I*_0_(*z*) and integration along *z*. Using (5) to fit the recorded I-QEPAS profile of CO, a ratio *I*_0_/*I*_sat_ = 22 was found to best describe the measured I-QEPAS profile of CO. The corresponding value *I*_sat_ = 1.2 W mm^−2^ found by inserting *I*_0_ = 26 W mm^−2^ agrees well with the saturation intensities given below. Inserting these values in (5) shows that the peak absorption coefficient is reduced to 0.14 *α*_0_.

### Measurement of saturation intensity

**Fig. 5:**
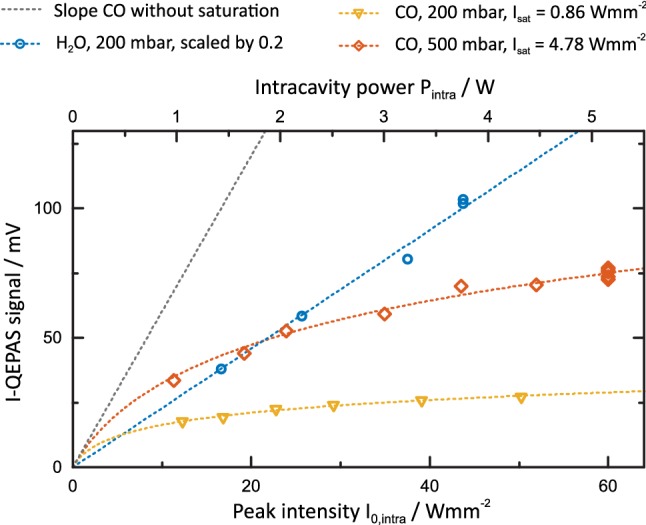
Scaling of I-QEPAS signals from CO (190 ppb) and H_2_O (1.7%_V_) with optical power. While H_2_O shows the expected linear behavior, strong saturation is observed for CO

To confirm that saturated absorption is the source of the observed line-broadening and to quantify the degree of saturation of CO in the cavity more accurately, the saturation intensity *I*_sat_ was determined using the I-QEPAS signal recorded at varying laser power. The scaling of the I-QEPAS signal with power was investigated for 190 ppb CO in N_2_ and 1.7%_V_ H_2_O at 200 mbar and 500 mbar, respectively. To this end, the beam was attenuated before entering the cavity via an iris and the optical power *P*_0_ was measured using a power meter. The intracavity power was obtained by multiplying *P*_0_ by the buildup factor and coupling efficiency retrieved in Sect. 2.3. Figure 5 shows the recorded I-QEPAS amplitude as a function of intracavity power and intensity. The background signal from water vapor was subtracted for each data point.

While the signal from H_2_O is directly proportional to optical power, the recorded signals from CO show a strong influence of optical saturation. The observed trend of I-QEPAS signal *S* with power can be described quantitatively based on (1). Inserting (5) and

6 $$\begin{array}{*{20}c} {P = \frac{\pi }{2}\sigma_{0}^{2} I_{0} } \\ \end{array} ,$$

[44] in (1) yields

7 $$\begin{array}{*{20}c} {S\left( {I_{0} } \right) = k\alpha_{0} \frac{\pi }{2}\sigma_{0}^{2} I_{\text{sat}} \ln \left( {1 + \frac{{I_{0} }}{{I_{\text{sat}} }}V\left( {\nu - \nu_{0} } \right)} \right).} \\ \end{array}$$

Herein, *α*_0_ is the 1/*e*^2^ beam radius, which equals 0.234 mm in the center of the cavity. For the measurements discussed herein and in the following chapters, the peak normalized line-shape function *V* = 1 since the wavelength was kept at the absorption peak.

To fit the experimental data using (7) and retrieve *I*_sat_, the factor *k* had to be determined separately rather than including it as a fitting parameter together with *I*_sat_. Fitting of both parameters together yielded unsatisfyingly large confidence intervals due to the large collinearity of *I*_sat_ and *k*. The factor *k* was determined using 2*f*-WM QEPAS measurements with known optical power *P*_0_ and absorption coefficient *α*_0_ and the same beam radius *σ*_0_ = 0.234 mm. The 2*f*-WM QEPAS signal is given by:

8 $$\begin{array}{*{20}c} {S_{{ 2f{\text{QEPAS}}}} = \frac{0.342}{0.5}kP_{0} \alpha_{0} .} \\ \end{array}$$

The pre-factor accounts for the different amplitudes observed in 2*f*-wavelength modulation and intensity modulation. The factor 0.342 corresponds to the Fourier component of a peak normalized Lorentzian profile at the second harmonic and optimum modulation amplitude at resonance [45]. The factor 1/0.5 corresponds to the fraction of intracavity optical power in the first harmonic as determined from experiment (compare Fig. 2). With *k*_500mbar_ = 2.7 × 10^9^ V cm W^−1^ and *k*_200mbar_ = 3.5 × 10^9^ V cm W^−1^ determined from 2*f*-WM QEPAS measurements of 20 ppm of CO, fitting of the data in Fig. 5 yielded saturation intensities of 4.78 [4.57; 4.98] W mm^−2^ and 0.86 [0.84; 0.88] W mm^−2^ for *p* = 500 mbar and *p* = 200 mbar, respectively (90% confidence interval in angled brackets).

From the fit of experimental data, the expected signal in absence of saturation (*I*_sat_ → ∞) can be calculated (compare Fig. 5, slope without saturation). The photoacoustic signals measured for maximum optical power and *p* = 200 mbar and *p* = 500 mbar are strongly influenced by saturation and correspond to 5.2% and 18%, respectively, of the calculated non-saturated signal. Assuming an effective two-level model for CO analogous to [43], the population *ρ*_A_ of the vibrational ground state can be estimated (compare (32) in [43]). For *I*_sat_ (500 mbar) = 4.78 W mm^−2^ and *I*_0_ = 73 W mm^−2^, *ρ*_A_ in the center of the beam (*r* = 0) and at *r* = *σ*_0_ is depleted to 53% and 66%, respectively, of its equilibrium value, close to the fully depleted value of 50%.

### CO calibration

**Fig. 6:**
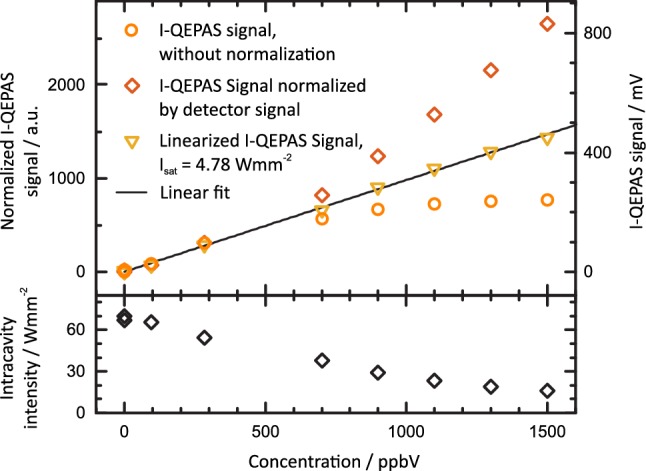
Calibration of CO in humidified N_2_ at 500 mbar. For high concentrations, strong absorption leads to a decrease in power buildup and reduced I-QEPAS signal (circles). On the contrary, the power normalized I-QEPAS signal’s slope d*S*/d*c* increases at high concentrations due to the absorption related decrease of intensity (lower panel) and hence weaker optical saturation. By correcting for both, intracavity power and saturation using (9), a linear calibration is obtained

To investigate the influence of saturation on the retrieval of CO concentrations and to characterize the performance of the setup, a series of calibration measurements was performed. For these measurements, the laser wavelength was locked to a cavity resonance and the cavity resonance wavelength was tuned to the peak of the absorption line of CO at 2179.77 cm^−1^. Measurements were performed at 500 mbar and the concentration *c* of CO in N_2_ and 1.7%_V_ H_2_O was varied from 0 ppb to 1500 ppb (*c* between 300 and 700 ppb could not prepared from the available gas standards). The integration time per data point was 120 s. The results are shown in Fig. 6.

For *c* > 300 ppb, the I-QEPAS signal’s sensitivity d*S*/d*c* decreases (circles in Fig. 6). This is due to a decrease in intracavity power *P*_intra_ caused by absorption by CO [38] [increasing losses *αL* in (2)]. To correct for this, *P*_intr*a*_ was measured for each concentration using the demodulated detector signal and the known value *P*_intra_ = 6.3 W for *c* = 0 and the results are shown in Fig. 6. Normalization by the demodulated detector signal, which corresponds to *P*_intra_, yields an increase in sensitivity of the normalized I-QEPAS signal at high concentrations (diamonds in Fig. 6). This is due to the decrease of the degree of optical saturation *I*_0_*/I*_sat_ and corresponding increase of *α* [compare (7)] with decreasing intracavity intensity *I*_0_. Based on (7), the recorded normalized QEPAS amplitude *S*_norm_(*c*) can be linearized using:

9 $$\begin{array}{*{20}c} {S_{\text{norm,lin}} \left( c \right) = S_{\text{norm}} \left( c \right)\frac{{I_{0} \left( c \right)}}{{\ln \left( {1 + \frac{{I_{0} \left( c \right)}}{{I_{\text{sat}} }}} \right)}}.} \\ \end{array}$$

Figure 6 shows the scaled linearized QEPAS amplitude *S*_norm,lin_ calculated for *I*_sat_ = 4.78 W mm^−2^ as retrieved in Sect. 3.2.

**Fig. 7:**
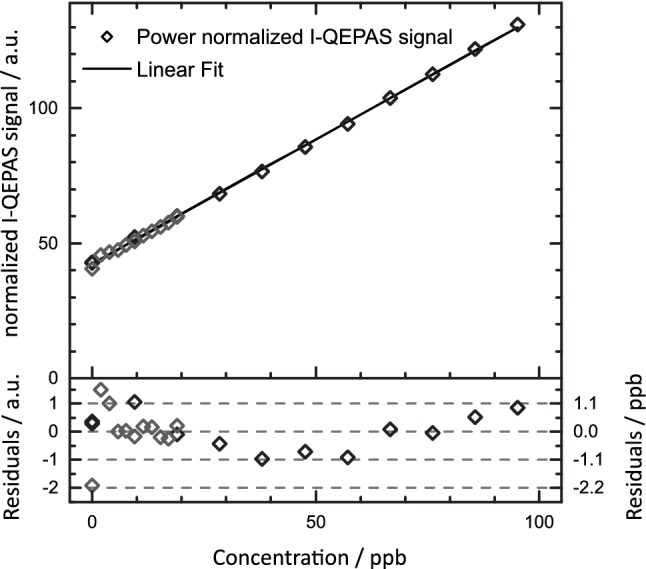
Calibration of CO in humidified N_2_. Grey and black data points correspond to measurements performed on different days

To demonstrate the capability of the setup for high sensitivity measurements of CO in a strongly saturated regime (*I*_0_ = 73 W mm^−2^ = 15 *I*_sat_), calibration curves were recorded from 0 to 95 ppb and 0 to 19 ppb (compare Fig. 7, experimental conditions identical to Fig. 6). For these small concentrations, no linearization of normalized I-QEPAS signals was necessary since *I*_0_ does not change sufficiently to influence *α*. The background signal at 0 ppb originates from the tail of the absorption line of water at 2178.90 cm^−1^.

### Sensing performance

**Fig. 8:**
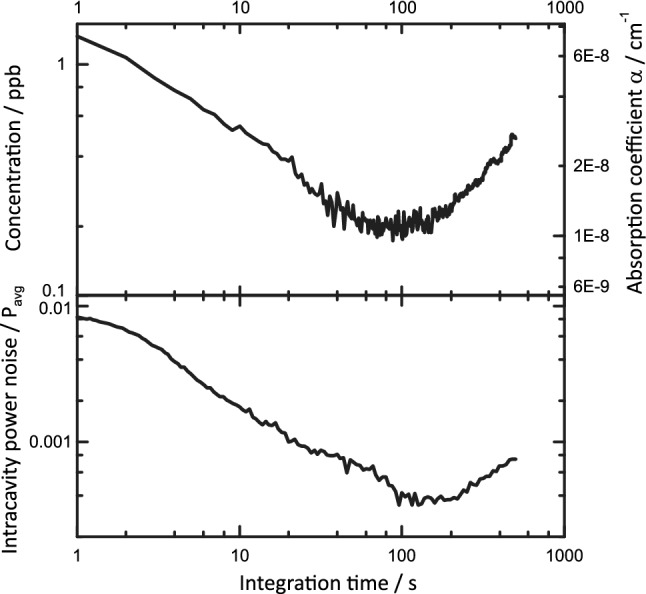
Allan–Werle plot recorded for humidified N_2_. Top: Allan deviation of normalized QEPAS signal, expressed as the concentration of CO (compare Fig. 7) and corresponding peak absorption coefficient *α* of CO at 2179.77 cm^−1^. Bottom: Allan deviation of demodulated detector signal, representing intracavity power

To characterize the sensing performance of the setup under saturated absorption as well as under linear absorption conditions we use the absorption lines of CO (2179.77 cm^−1^) and H_2_O (2178.90 cm^−1^) and compare the results obtained for I-QEPAS with those of 2*f*-WM QEPAS. The noise floor of both QEPAS measurements is determined from Allan variance analysis. An Allan–Werle plot [46] recorded at conditions identical to the calibrations presented above, i.e. 1.7%_V_ water in N_2_ at 500 mbar, in the absence of CO is shown in Fig. 8. The noise floor up to ~ 80 s integration of 0.76 mV Hz^−1/2^ was dominated by noise of the tuning fork (thermal noise) and was identical for I-QEPAS and 2*f*-WM QEPAS of both, H_2_O and CO. Additional potential drifts, limiting the Allan deviation at integration times larger than 100 s, may arise from small changes in water concentration, and hence background signal, and from temperature changes affecting the resonance frequency of the tuning fork.

From the calibration shown in Fig. 7, the sensitivity for CO detection is 0.369 mV ppb^−1^ for I-QEPAS and 6.64 × 10^−3^ mV ppb^−1^ for 2*f*-WM QEPAS (calibration not shown). From a one-point calibration, the sensitivity of I-QEPAS and 2*f*-WM QEPAS for H_2_O was found to be 29.7 µV ppm^−1^ and 0.13 µV ppm^−1^, respectively. The corresponding noise equivalent concentrations (NEC), noise equivalent absorptions (NEA) and power normalized NEAs (NNEA) of I-QEPAS and 2*f*-WM-QEPAS are summarized in Table 1. Also, the gain in signal to noise ratio (SNR) from I-QEPAS as compared to 2*f*-WM QEPAS is given. The figures of merit for CO detection are adversely influenced by optical saturation as well as slow *V*–*T* relaxation [4, 47]. However, despite strong optical saturation, the sensitivity is increased by a factor of 57 as compared to 2*f*-WM QEPAS without power enhancement. The full potential of cavity enhancement for PAS in the absence of optical saturation is evident from the gain of 230 in SNR obtained for H_2_O, which agrees well with the power enhancement of about 250 found above.

**Table 1:** Figures of merit of I-QEPAS compared with 2*f*-WM QEPAS for sensing of CO and H_2_O

Unit	NEC (ppb Hz^−1/2^)		NEA (10^−6^ cm^−1^ Hz^−1/2^)		NNEA (10^−9^ cm^−1^ W Hz^−1/2^)		SNR gain	
	CO	H_2_O	CO	H_2_O	CO	H_2_O	CO	H_2_O
I-QEPAS	2.1	2590	0.11	0.0044	2.8	0.07	57	230
2*f*-WM QEPAS	114	5.8 × 10^6^	6.3	0.99	160	16		

NEA and NNEA are calculated from NEC using NEA(CO) = NEC × 5.5 × 10^−8^ cm^−1^ ppb^−1^, NEA(H_2_O) = NEC × 1.7 × 10^−13^ cm^−1^ ppb^−1^; NNEA(CO) = NEA × 25 mW, NNEA(H_2_O) = NEA × 16 mW

From the same measurement of humidified N_2_ discussed above, an Allan variance analysis of the demodulated detector signal, normalized by its mean value, was performed (see Fig. 8, bottom panel). This represents the noise of the intracavity power *P* and translates directly to the relative uncertainty of a measurement in the absence of all other noise sources [compare (1)]. The noise floor of *P*/*P*_avg_ of 5.7 × 10^−3^ Hz^−1/2^ is large compared to the laser noise, but low compared to typical measurements with high finesse cavities and free-running lasers [19]. The low intracavity power noise is a distinct feature of the optical-feedback locking technique and is commonly exploited in optical feedback cavity-enhanced absorption spectroscopy (OF-CEAS) [9].

## Conclusions

The presented work demonstrates highly sensitive cavity-enhanced QEPAS measurements of CO under strong optical saturation. Optical feedback from a Brewster window cavity was used to achieve low noise intracavity power enhancement by a factor of 252, corresponding to a power of 6.3 W and intensity of 73 W mm^−2^, respectively, which yielded strongly saturated photoacoustic signals. Theory describing photoacoustic signals under saturated absorption in a Gaussian beam was presented. On this basis, the saturation intensity of the R9 transition of CO in humidified N_2_ at 500 mbar and 200 mbar was retrieved from the power dependence of the I-QEPAS signal. This allowed linearization of a calibration curve affected by varying degrees of optical saturation, which was caused by changes in optical power inside the high finesse cavity. The presented quantitative description of photoacoustic signals under saturated absorption allows making full use of the sensitivity enhancement in optical cavities beyond the linear regime. As an example, assuming one would limit the intensity to a linear regime, e.g. to $I_{0} < \frac{{I_{\text{sat}} }}{50}$ such that *α* > 0.99 *α*_0_ [compare (5)], the sensitivity would be ~ 140 times smaller than that obtained for *I*_0_ = 73 W mm^−2^ and *I*_sat_ = 4.78 W mm^−2^, corresponding to the measurements presented above [compare (7)]. Therefore, despite the necessity of correcting for the non-linear sensor response, operating in the non-linear regime significantly improves the sensitivity.

The sensitivity of I-QEPAS measurements of CO and H_2_O was compared to 2*f*-WM QEPAS measurements without power enhancement. For the non-saturated transition of H_2_O, the expected increase in SNR of 230 was found, very close to the power enhancement. Although the gain in sensitivity for CO is limited by saturation, the SNR was increased by a factor of 57, demonstrating that the sensitivity of PAS can be pushed beyond the linear range.

Future work will focus on improving the QEPAS detection, e.g. using optimized resonator tubes and tuning-forks, to lower detection limits [2]. Also, the volume of the gas cell can be drastically reduced using the Brewster window as a window of the gas cell. Exploiting optical feedback, light can be efficiently coupled into cavities of significantly higher finesse than presented herein, yielding even higher intracavity powers [38]. The resulting intracavity intensities exceed the saturation intensities of many molecules. As demonstrated on the example of CO, correcting PAS signals for saturated absorption is feasible and allows increasing the sensitivity beyond the linear regime.

## Figures and Tables

**Fig. 9:**
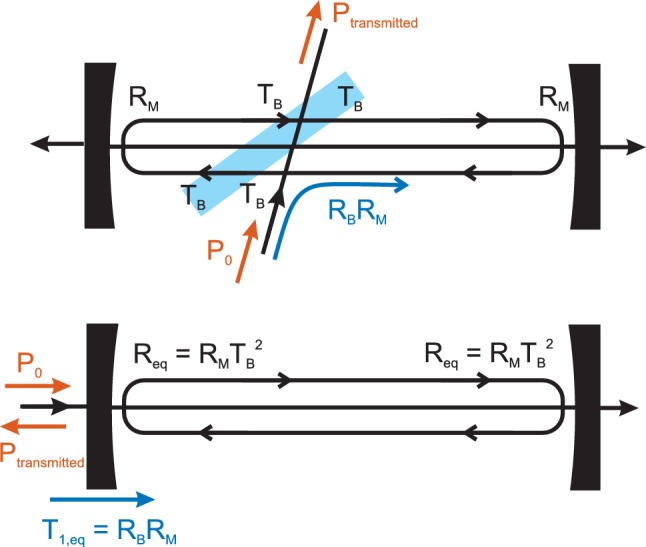
Losses (black) and gain from coupling of light into the cavity (blue) in the Brewster window cavity (top) and the equivalent linear two-mirror cavity (bottom)

## Appendix A: Optical power buildup in a Brewster window cavity

The power *P*_intra_/*P*_0_ and cavity reflectivity *R*_cav_ on resonance were derived previously for the linear two mirror cavity [48, 49]:

10 $$\begin{array}{*{20}c} {P_{\text{intra,lin}} = P_{0} \frac{{2T_{1} }}{{\left( {1 - R_{\text{M}} + \alpha L} \right)^{2} }}} \\ \end{array}$$

11 $$\begin{array}{*{20}c} {R_{\text{cav}} = \frac{{P_{\text{reflected}} }}{{P_{0} }} = \frac{{\left( {1 - R_{\text{M}} + \alpha L - T_{1} } \right)^{2} }}{{\left( {1 - R_{\text{M}} + \alpha L} \right)^{2} }}} \\ \end{array} .$$

Herein, *T*_1_ is the transmission of the in-coupling mirror and 2*αL* are all other round-trip losses of the cavity (typically gas absorption). The reflectivity *R*_M_ of both mirrors is assumed equal.

To derive corresponding expressions for *P*_intra_ and the transmission $T_{\text{Brewster}} = \frac{{P_{\text{transmitted}} }}{{P_{0} }}$ of the Brewster window cavity, a linear cavity equivalent to the Brewster window can be defined (see Fig. 9). To this end, the reflective losses at the Brewster window are included in the mirror reflective losses, $R_{\text{M,eq}} = R_{\text{M}} T_{\text{B}}^{2} \approx R_{\text{M}} - 2R_{\text{B}}$, and the transmission of the in-coupling mirror is chosen $T_{{ 1 , {\text{eq}}}} = R_{\text{B}} R_{\text{M}} \approx R_{\text{B}}$. The first approximation holds for small *R*_B_ and $R_{\text{B}} = 1 - T_{\text{B}}$, the latter approximation holds for *R*_M_ ≈ 1. Further, we realize that, due to the inverted role of reflection and transmission of the Brewster window as compared to a cavity mirror, *T*_Brewster_ equals the reflectivity of the equivalent two-mirror cavity *R*_cav,eq_. Substituting *T*_1,eq_ for *T*_1_ and *R*_M,eq_ for *R*_M_ in (10) and (11) yields (2) and (4), respectively. Non reflective losses at the Brewster window can be included in (2) and (4) by adding a term equivalent to *αL* in the denominator.

## Appendix B: Properties of the Brewster window cavity for intracavity PAS

The Brewster window cavity, as compared to all-mirror cavities, features some interesting properties for intracavity PAS. First, the volume of the gas cell can be decreased drastically using the Brewster window as a window of the gas cell. Hence, a small sample volume can be enclosed by a cavity mirror and the Brewster window, and the cavity length can be chosen independently from the sample volume. For all-mirror cavities, miniaturization can only be achieved by reducing the cavity length, which increases the free spectral range. Second, in a single cavity, the finesse and power buildup can be varied by orders of magnitude by adjusting the window’s angle (compare Sect. 2.3) [38] or by changing the polarization to *s*, e.g. via a half-wave plate. This holds promise to drastically increase the dynamic range of intracavity measurements. Third, using a Brewster window for coupling light into a cavity allows using cavity mirrors designed for maximum reflectivity without taking care of their transmission. For mirrors of very high reflectivity, the transmission *T*_1_ can become much smaller than the mirror losses 1 − *R*_M_ + *αL* (here, *αL* denotes scattering and absorption losses of the mirror), which limits the power buildup in all-mirror cavities [compare (10)]. Whether or not the Brewster window can improve on this situation depends on the non-reflective losses at the Brewster window as compared to those of the mirror. Non-reflective losses at the Brewster window originate from scattering, absorption and reflection of s-polarized components of the cavity mode. In the mid-IR, scattering is negligible if polished substrates are used. Absorption is strongly wavelength dependent and can be reduced using thin windows. At *λ* = 4.5 µm, the absorption coefficient of CaF_2_ is ~ 3 × 10^−4^ cm^−1^. Reflection of s-polarized light may occur due to polarization rotation caused by mirror birefringence. Polarization rotation can be minimized by rotating the mirrors against each other [50].
